# Recurrent and metastatic female adnexal tumor of probable Wolffian origin: A case report and review of the literature

**DOI:** 10.1097/MD.0000000000025377

**Published:** 2021-04-02

**Authors:** Qiuhe Chen, Yangmei Shen, Chuan Xie

**Affiliations:** a Department of Gynecology and Obstetrics, West China Second University Hospital, Sichuan University; b Key Laboratory of Birth Defects and Related Diseases of Women and Children, Ministry of Educationa; c Department of Pathology, West China Second University Hospital, Sichuan University, Chengdu, Sichuan Province, P.R. China.

**Keywords:** chemotherapy, female adnexal tumor of probable Wolffian origin, management, prognosis

## Abstract

**Rationale:**

Female adnexal tumors of probable Wolffian origin (FATWOs) are rare gynecologic neoplasms arising from the mesonephric duct remnants. Less than 90 cases have been reported in the English literature. Although most cases of FATWO are considered benign, recurrence and metastasis may occur in very few cases during the course of the disease. Due to the small number of recurrent and metastatic FATWO cases, there are no clear recommendations regarding optimal treatment.

**Patient concerns:**

A 75-year-old postmenopausal woman, who underwent a mass excision of the right broad ligament three years ago, was found to have a right adnexal mass during a regular postoperative physical examination.

**Diagnoses:**

Vaginal ultrasound examination revealed a cystic and solid mass approximately 3.6 × 4.4 × 3.8 cm on the right side of the uterus. Three years ago, the mass of the right broad ligament was diagnosed with FATWO in the local hospital. Following extensive immunohistochemistry analysis and after reviewing the histology slides from the primary tumor, the final diagnosis of the mass on the right side of the uterus was recurrent and metastatic FATWO.

**Interventions:**

The patient underwent laparoscopic mass excision, hysterectomy and resection of the metastatic lesion in the small intestine, and then she received 6 cycles of docetaxel and carboplatin-based chemotherapy.

**Outcomes:**

The disease has recurred three years after the first surgery in the local hospital. After the second surgery followed by systemic chemotherapy, there is no evidence of recurrence with 24 months of follow-up till now.

**Lessons:**

FATWO is considered a benign entity. However, a few FATWOs have been shown to behave aggressively. Due to only a few reported cases, there are no comprehensive recommendations regarding the optimal clinical management of recurrent and metastatic FATWOs. Complete surgical resection followed by combination chemotherapy is considered to be the most effective therapy for recurrent and metastatic FATWOs. Chemotherapy with docetaxel plus carboplatin, which is most commonly used in malignant cases, may be effective in the treatment of recurrent and metastatic FATWOs.

## 1 Introduction

Female adnexal tumors of probable Wolffian origin (FATWOs) are rare gynecologic neoplasms derived from mesonephric duct remnants of the female genital tract, with >90 cases reported in the English literature.^[1]^ FATWOs were first reported by Kariminejad and Scully in 1973, and they regarded the tumor as a nonmalignant neoplasm despite the presence of mitotic activity and capsular invasion.^[2]^ These tumors are considered to have low malignant potential over time, as only a few cases have been found to relapse and metastasize and most of these tumors have a benign course.^[3]^ It has been reported that recurrence and metastasis occur in approximately 11% of cases and they may occur as early as 2 years after diagnosis.^[3]^ Due to the few reported cases, there are no clear recommendations regarding the optimal management of recurrent and metastatic FATWOs. Here, we report a case of recurrent and metastatic FATWO arising from the broad ligament and review the literature on the optimal management and prognosis of this disease, to provide clinicians with a better understanding and management of the disease.

## 2 Case presentation

Ethical approval and patient consent were acquired and recorded in the patient medical record with witness signature. All ethical approval and consent procedures were approved by the Medical Ethical Committee of West China Second University Hospital, Sichuan University.

A 75-year-old post-menopausal G3P2 Chinese woman originally presented in 2015 with a right adnexal mass. At laparotomy, she was noted to have a mass approximately 8 cm in diameter arising from the broad ligament. There was no evidence of any other pelvic or abdominal disease. The patient underwent a simple mass excision combined with prophylactic bilateral adnexectomy in a local hospital. After consultation with two different external expert pathologists, the mass was suggested to be a FATWO. The patient had a good recovery without complications. In October 2018, vaginal ultrasound at our institution during routine follow-up showed a cystic and solid mass approximately 3.6 × 4.4 × 3.8 cm in the right adnexal area. The patient did not have vaginal bleeding, abdominal pain, abdominal distension or other discomfort. There was no family history of breast or ovarian cancer. On physical examination, except for the mass on the right side of the uterus, no other abnormalities were recorded. Laboratory test results, including blood cell counts and tumor markers such as cancer antigen-125, were all within the normal ranges. Pelvic computed tomography (CT) suggested a cystic and solid mass about 5 × 4 cm on the right side of the uterus, with a regular shape and close contact with the wall of the uterus.

After a detailed explanation and comprehensive counseling regarding the advantages of a single surgical intervention, laparoscopy was performed. At diagnostic laparoscopy, gross examination showed that there was a solid and cystic mass approximately 5 × 4 cm in size on the right side of the pelvic cavity, which was densely adhered to the posterior leaf of the right broad ligament, the right posterior lateral wall of the uterus and the small intestine. Intraoperative findings showed no obvious ascites fluid. Except for the right posterior lateral wall of the uterus, the posterior leaf of the right broad ligament and part of the small intestine, no obvious tumor invasion was observed in other sites of the abdominal and pelvic cavity. Resection of the tumor mass combined with a total hysterectomy was performed, and peritoneal washing was also undertaken during surgery. The postoperative course was uneventful and the patient was discharged on day 5 after surgery.

**Figure 1:**
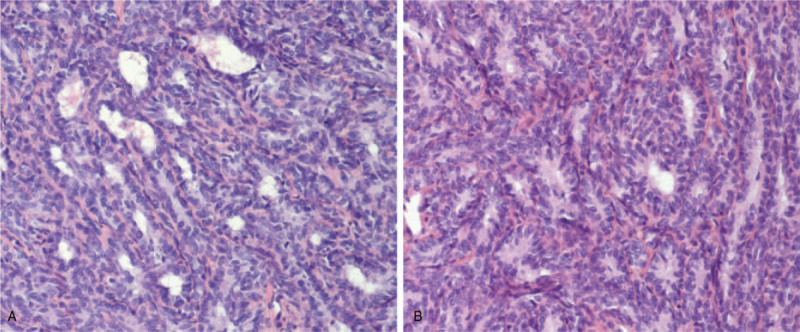
Histologic features of the patient with FATWO. (A, B)The histologic pattern of the tumor was arranged in a glandular manner: the glandular tubes were densely arranged, curved, and branch anastomoses with each other, and they were covered by cubic or columnar epithelial cells. (A) Some areas of the tumor included cystic changes. (B) Some of the glands contained luminal eosinophilic material (hematoxylin-eosin [HE] staining, original magnification ×200).

Gross examination of the excised specimen showed that the tumor mass was mainly solid and partly cystic, and the cut surface of the solid component was gray-white. Pathological findings revealed that the neoplastic cells were diffuse shaped by solid, tubular, and sieve-like pattern, with some cystic changes. Local areas of fibrous stroma divided the tumor into lobules. Most of the cells were square or columnar; the round or oval nuclei were cytologically bland with fine, evenly dispersed chromatin, an absence of discernible nucleoli and a low mitotic index (Fig. 1). Immunohistochemical stainings for CK7, Vimentin, EMA, TTF-1, CD10, Pax-8, Pax-2, P16, and CA125 were positive and ER, PR, AR, CEA, α-inhibin, CD56, and CK20 were negative. The Ki-67 index of the tumor was around 60%. Following extensive immunohistochemistry analysis and reviewing the histology slides from the primary tumor, the final diagnosis was recurrent and metastatic FATWO. According to the current ovarian cancer staging system of the International Federation of Gynecology and Obstetrics (FIGO, 2017), this tumor was staged as IIIC.

A medical oncologist was consulted, and additional chemotherapy was recommended. The patient received adjuvant chemotherapy with 6 cycles of docetaxel (80 mg/m^2^) and carboplatin (300 mg/m^2^) 3 weeks after the second surgery. The patient was regularly reviewed for chest and abdominal CT scans and tumor markers; there was no evidence of recurrence at 2 years after the second surgery. We will continue to follow-up this patient.

## 3 Discussion

FATWOs are extremely uncommon tumors with low malignant potential and are believed to derive from mesonephric remnants. To date, approximately 90 cases of FATWO have been reported. Although most cases of FATWO exhibit a benign clinical behavior, a few cases have been shown to be aggressive. In recent years, some metastatic and recurrent cases have been reported. We searched the PubMed and GeenMedical databases using the keywords “Wolffian origin,” “malignant,” and “metastatic,”, and found 26 studies published between 1976 and 2017.^[1,4–28]^ These studies involved 28 patients (Table 1). According to the results in Table 1, women of all ages are likely to develop malignant FATWO, and the age of the patients diagnosed with FATWO ranged from 15 to 81 years. However, most patients were aged >50 years at the time of initial diagnosis. FATWO mainly arises in the broad ligament and occasionally occurs in the mesosalpinx, ovary, and fallopian tube, but rarely occurs in the retroperitoneum and paravaginal region. The omentum and peritoneum are the most frequent metastatic sites, and other common metastatic sites are the liver, bowel serosa, lung, and paravaginal region. The median time to recurrence and metastasis was 33.5 months with a range of 4 to 96 months. Most recurrent and metastatic diseases developed in patients who were initially treated with tumor resection alone.

**Table 1:** Reported metastatic or recurrent cases of FATWO.

Case	Author	Age, y	Primary site	Operation	Stage	Treatment after surgery	Site of metastasis or recurrence	Treatment after recurrence	Follow-up
1	Taxy and Battifora, 1976^[1]^	41	Right Broad ligament	1. H, BSO 2. Abdominal exploration	I	Pelvis cobalt Irradiation	Liver	NA	METS, 6 y
2	Buntine, 1979^[4]^	NA	Vaginal apex	H, BSO	NA	Radiotherapy	NA	NA	NED, 15 mo
3	Abbott et al, 1981^[5]^	18	Right mesosal-pinx	1. STR 2. BSO, omentectomy 3. Tumor implants removal	I	No	1. Pelvis and peritoneum 2. Peritoneum, mesenteries, bowel serosa, and the left hemidiaphragm	1. Cyclophosphamide, doxorubicin, and cis-platinum 2. 5-FU, peptichemio and bleomycin	1. METS, 6 y 2. METS, 1 y 3. DOD, 1 y
4	Hughesdon, 1982^[6]^	79	Left ovary	BSO	I	No	Pouch of Douglas	NA	DOD, 1 y
5	Young and Scully, 1983^[7]^	64	NA	BSO, omentectomy, subtotal colectomy	III	NA	Colonic serosa	NA	LFU
6	Young and Scully, 1983^[7]^	52	NA	H, BSO	IA1	NA	Bilateral lung	NA	METS, 8 y
7	Brescia et al, 1984^[8]^	23	Retroperitoneal mass	1. STR 2. Mass resection 3. Right hepatectomy	I	No	1. Omentum, bowel serosa, retroperitoneal mass 2. Omentum at the hepatic flexure 3. Right liver	1. Pelvis radiation 2. Abdomen radiation	1. REC, 21 mo 2. REC, 3 y 3. REC, 7 y 4. NED, 5 y since the third recurrence.
8	Prasad et al, 1992^[9]^	47	NA	NA	III	NA	Peritoneum	NA	NA
9	Daya et al, 1993^[10]^	20	Right paravaginal mass	1. STR 2. Small biopsy 3. Laparotomy	I	No	1. Previous surgery site 2. Right paravaginal areas	1. Radiotherapy and cisplatin	1. REC, 2 y 2. REC, 3 y
10	Daya, 1994^[11]^	81	Right broad ligament	NA	NA	NA	Extensive omentum	NA	DOD, 3mo
11	Sheyn et al, 2000^[12]^	60	Right broad ligament	H, BSO, omentectomy, appendectomy	III	Cisplatin-cytoxan chemotherapy	Right liver	NA	METS, 5-y
12	Ramirez et al, 2002^[13]^	38	Right paratubal nodule	1. H, nodule removal 2. Mass resection, BSO, omentectomy, perihepatic masses excision and appendectomy	I	No	1. Pelvic mass, omental nodule and perihepatic implants 2. Liver, spleen, pelvis	1. Carboplatinum and paclitaxel and one dose of intra-muscular leuprolide	1. METS, 3 y 2. METS, 4 mo
13	Ramirez et al, 2002^[13]^	71	Pelvis	Before: H, BSO Lately: omentectomy, and tumor reductive surgery	III	No	1. Bowel mesentery, and omentum 2. Peritoneal implant, liver	NA	METS, 10 mo
14	Halushka and Ali, 2004^[14]^	34	Right fallopian tube	1. BSO 2. Debulking procedure, H	NA	No	Right groin	Chemotherapy	METS, 2 y
15	Atallah et al, 2004^[15]^	27	Left broad ligament	1. STR 2. H, BSO, omentectomy, pelvic and para-aortic lymph node dissection	I	No	Multiple peritoneal implants	Cisplatin and cyclophosphamide; paclitaxel and cisplatin	1. METS, 3 y after pregnancy; 2. DOD 5 y
16	Steed et al, 2004^[16]^	15	Rght broad ligament	1. STR 2. Tumor removal 3. Debulking surgery 4. H, BSO, upper vaginectomy, large and small bowel resections, and debulking surgery	I	No	1. Right broad ligament and left abdominal wall. 2. Abdominal wall and pelvic masses 3. Broad ligament 4. Right liver	1. Cisplatin and cyclophosphamide 2. Amifostine, etoposide, ifosfamide, and carboplatin; irinotecan 3. Epothilone B; Gleevac	1.METS, 2 y 2. METS,1 y3.3.METS, 1 y 4. NED, 10 mo
17	Sivridis et al, 2005^[17]^	76	Right broad ligament	H, BSO	III	No	Peritoneum	No	DOD, 4 mo
18	Tamiolakis and Anastasiadis, 2006^[18]^	75	Right ovary	A right ovary and broad ligament resection	NA	Cisplatin–cytoxan chemotherapy	Left broad ligament	NA	METS, 2 y
19	Deen et al, 2007^[19]^	81	Right ovary	H, BSO, omentectomy	I	No	Right adnexa	No	NED, 7mo
20	Lesin and Forko-Ilic, 2009^[20]^	60	Right adnexa	1. H, BSO, omentectomy 2. Tumor mass excision	IA	No	Vaginal cuff	No	1. METS, 6 y 2. NED, 2 y
21	Syriac et al, 2011^[21]^	38	Right broad ligament	1. STR 2. H, BSO, lymphadenectomy, omentectomy and bilateral pelvic and para-aortic lymph node dissection	I	No	Left ovary	Gleevec	1. METS, 3 y
22	Liu, 2011^[22]^	24	Left broad ligament	1. STR 2. exploratory surgery	III	NA	1. Omentum 2. Appendix	N	NA
23	Deshimaru et al, 2014^[23]^	30	Right fallopian tube	1. USO, tumorectomy 2. H, BSO, omentectomy, tumorectomy and pelvic and para-aortic lymph nodes biopsies	NA	1. Paclitaxel and carboplatin 2. Irinotecan and gemcitabine	1. Bowel serosa, omentum, and left ovary. Pouch of Douglas 2. Abdominal cavity, included liver		1. METS, 4 mo 2. DOD, 3 y
24	Nakamura et al, 2014^[24]^	69	NA	NA	NA	N	NA	NA	RECR, 1 y
25	Kwon et al, 2016^[25]^	26	Left ovary	H, BSO, omentectomy and pelvic lymph node dissection	I	No	1. Vaginal stump 2. Liver	Paclitaxel and carboplatin	1. METS, 9 mo 2. LFU
26	Hong et al, 2017^[26]^	50	Bilateral ovaries	H, BSO, omentectomy, pelvic and para-aortic lymph node dissection	NA	NA	Uterine serosa	NA	NA
27	Qiu et al, 2017^[27]^	53	Left mesosalpinx	1. H, BSO, tumor resection and omentectomy 2. Pelvic masses resection and partial omentectomy	NA	No	1. Omentum, mesentery, and peritoneum 2. Right lung, right liver and left adrenal gland	1. Cisplatin and docetaxel; oxaliplatin and docetaxel 2. Continuous renal replacement and hepatoprotection therapy	1. METS, 2 y 2. METS, 2 mo 3. DOD at 83 days after the second surgery.
28	Wakayama et al, 2017^[28]^	37	Left fallopian tube	1. USO, pelvic lymph node sampling and omental biopsy 2. H, USO, tumor excision 3. Debulking surgery	II	No	1. Peritoneum 2. Douglas pouch, the right para-colic gutter and the hepatorenal fossa 3. Tumor dissemination	1. Glivec 2. Paclitaxel and carboplatin	1. METS, 1 y 2. METS, 6 wk 3. RECR, 6 wk
29	Present case	75	Right broad ligament	Before: BSO Lately: H, intestinal adhesion lysis	III	Docetaxel and carboplatin	Bowel serosa	No	NED, 2 y

BSO = bilateral salpingo-oophorectomy, DOD = dead of disease, H, BSO = hysterectomy with bilateral salpingo-oophorectomy, LFU = lost to follow-up, METS = metastasis, NED = no evidence of disease, RECR = recurrence, STR = simple tumor resection, USO = unilateral salpingo-oophorectomy.

The clinical manifestations of FATWOs are varied; patients with FATWOs may have abdominal pain or irregular vaginal bleeding as their main symptoms, or feel a palpable mass in the abdomen when the tumor is large enough. Many patients are asymptomatic, and the tumors are found incidentally during imaging studies or laparoscopy for other gynecological disease. Histologically, FATWOs can exhibit a variety of growth patterns, including tubular, sieve-like, solid and diffuse in various combinations. The tubal lumen and sieve-like spaces often contain eosinophilic, colloid-like, PAS-positive substance.^[3]^ Hence, early diagnosis may be difficult. Sivridis et al^[17]^ combined the maglignancies described earlier and proposedsdiagnostic criteria for malignant FATWO: tumors >10 cm in diameter, obvious hypercellularity, capsular invasion, capsular rupture and verifiable tumor implants and metastases. Fortunately, most FATWOs exhibit a benign clinical behavior.

Due to the rarity of FATWO and the few reported cases, optimal management has not yet been established. However, complete tumor resection, including hysterectomy, bilateral salpingo-oophorectomy, and debulking surgery, is the preferred treatment for FATWO. Most tumor relapses occur in patients initially treated with conservative procedures such as cystectomy or simple tumor resection.^[12,13,20]^ Although there are some case reports of remission or partial remission following specific adjuvant therapy, the exact effect of radiotherapy, chemotherapy, hormone therapy, and molecular-targeting therapy on malignant FATWO remains to be clarified. Therefore, it is imperative to institute appropriate treatment strategies in patients with malignant FATWO.

Multiple chemotherapy regimens have been used to treat recurrent and metastatic FATWO, such as paclitaxel/carboplatin,^[23,25,28]^ cisplatin/cyclophosphamide,^[15,16]^ etoposide/ifosfamide/carboplatin,^[16]^ cyclophosphamide/doxorubicin/cis-platinum,^[5]^ and cisplatin/oxaliplatin/docetaxel^[27]^ (Table 1); however, the effects were not satisfactory. A recent study^[28]^ showed that carboplatin and paclitaxel combination therapy produced a good response in patients with recurrent and metastatic FATWO following the failure of imatinib treatment. Atallah et al^[15]^ reported a patient with progesterone receptor-positive FATWO with tumor recurrence after pregnancy. The patient received paclitaxel plus cisplatin chemotherapy, which induced temporary disease stabilization. Qiu et al^[27]^ reported a patient with recurrent FATWO who was treated with cisplatin/oxaliplatin/docetaxel chemotherapy, which resulted in side effects and the patient had recurrent disease 2 months after the second surgery and died ultimately. In our patient, a good response to platinum-based chemotherapy was observed without side effects. In addition, there was no evidence of recurrence at 2 years’ follow-up after the second surgery, indicating that combination chemotherapy with docetaxel plus carboplatin may be effective for treating recurrent and metastatic FATWOs. However, further studies are needed to determine the effectiveness of this chemotherapy regimen.

The prognosis of FATWO is independent of clinical presentation and histological features, and recurrence can still occur in the absence of aggressive histological findings.^[15]^ It was reported that the median recurrence time for FATWOs was 48 months with a range of 13 to 96 months, and liver and lung were the most frequent metastatic sites.^[13,20]^ According to the findings shown in Table 1, the median time to recurrence and metastasis was 33.5 months, ranging from 4 to 96 months, and some cases experienced multiple relapses during this time. The most common site of tumor metastasis is the omentum and peritoneum, followed by the liver, bowel serosa, lung and paravaginal region. Only 1 case metastasized to the appendix. The presence of necrosis, capsular invasion, a high number of mitoses, cellular pleomorphism, immunohistochemical positivity for CD117 and, probably, overexpression of Ki-67 are the currently known properties of FATWOs with malignant potential.^[1]^

## 4 Conclusions

FATWOs are rare gynecologic neoplasms of low-malignant potential which are considered to derive from mesonephric remnants. Although most cases of FATWO have a benign course, some have the potential for recurrence and metastasis, and a few patients have died of the disease within a short time. Due to only a few reported cases, there are no distinct recommendations regarding the optimal management of recurrent and metastatic FATWOs. We reviewed previous cases to determine the best treatment protocol. Complete surgical resection with hysterectomy, bilateral adnexectomy, and debulking of the tumor, followed by combination chemotherapy is considered to be the most effective therapy for recurrent and metastatic FATWOs. Chemotherapy with docetaxel plus carboplatin, which is most commonly used in malignant cases, may be effective in the treatment of recurrent and metastatic FATWOs.

## Author contributions

**Conceptualization:** Qiuhe Chen, Chuan Xie.

**Data curation:** Qiuhe Chen, Yangmei Shen, Chuan Xie.

**Formal analysis:** Qiuhe Chen.

**Investigation:** Qiuhe Chen, Chuan Xie.

**Methodology:** Yangmei Shen, Chuan Xie.

**Software:** Qiuhe Chen, Yangmei Shen, Chuan Xie.

**Supervision:** Qiuhe Chen, Chuan Xie.

**Writing – original draft:** Qiuhe Chen, Chuan Xie.

**Writing – review & editing:** Qiuhe Chen, Chuan Xie.
